# Diagnostic Performance of Des-*γ*-carboxy Prothrombin for Hepatocellular Carcinoma: A Meta-Analysis

**DOI:** 10.1155/2014/529314

**Published:** 2014-08-06

**Authors:** Rong Zhu, Jing Yang, Ling Xu, Weiqi Dai, Fan Wang, Miao Shen, Yan Zhang, Huawei Zhang, Kan Chen, Ping Cheng, Chengfen Wang, Yuanyuan Zheng, Jingjing Li, Jie Lu, Yingqun Zhou, Dong Wu, Chuanyong Guo

**Affiliations:** ^1^Department of Gastroenterology, Shanghai Tenth People's Hospital, Tongji University School of Medicine, Shanghai 200072, China; ^2^Department of Gastroenterology, Tongren Hospital, Shanghai Jiao Tong University School of Medicine, Shanghai 200050, China; ^3^Department of Gastroenterology, Ningbo No. 2 Hospital, Ningbo 315010, China

## Abstract

*Background*. There have been many reports on des-*γ*-carboxy prothrombin (DCP) as a promising serum marker in the diagnosis of hepatocellular carcinoma (HCC); however, the results are inconsistent and even conflicting. *Methods*. This meta-analysis was performed to investigate the performance of DCP in the diagnosis of HCC. Following a systematic review of relevant studies, Meta-DiSc 1.4 software was used to extract data and to calculate the overall sensitivity, specificity, positive likelihood ratio (PLR), negative likelihood ratio (NLR), and diagnostic odds ratio (DOR). Data are presented as forest plots and summary receiver operating characteristic curve (SROC) analysis was used to summarize the overall test performance. *Results*. Twelve studies were included in our meta-analysis. The overall sensitivity, specificity, PLR, and NLR of DCP for the detection of HCC in the studies included were 71% (95%CI: 68%–73%), 84% (95%CI: 83%–86%), 6.48 (95%CI: 4.22–9.93), and 0.33 (95%CI: 0.25–0.43), respectively. The area under the SROC curve was 0.8930 and the *Q* index was 0.8238. Significant heterogeneity was found. *Conclusion*. This meta-analysis indicated that DCP had moderate diagnostic accuracy in HCC. Further studies with rigorous design, large sample size, and mmultiregional cooperation are needed in the future.

## 1. Introduction

Hepatocellular carcinoma (HCC) is the most common primary liver malignancy and the third most common cause of cancer death worldwide [1]. Approximately 500,000 new cases of HCC are reported each year and more than 75% of cases occur in the Asia- Pacific region, largely in association with chronic hepatitis B virus infection [2, 3]. Each year an estimated 360000 patients living in the Far East countries (including China, Japan, and South Korea) die of liver cancer [4]. HCC usually develops in an already damaged liver, often in patients with cirrhosis. In most areas, chronic viral hepatitis caused by hepatitis B virus or hepatitis C virus is the major cause of HCC [5]. Usually, HCC is diagnosed at a late stage, and for these patients, the outcome of current medical treatments including chemotherapy, chemoembolization, ablation, and proton beam therapy is disappointing, with a 5-year survival rate of less than 5% [6]. Therefore, animal models of HCC should be established to facilitate research into the pathogenesis of HCC and to target therapies [7–9]. The detection of HCC at an early stage is very important. However, in most cases, early diagnosis of HCC is complex, because HCC is usually accompanied by inflammation and liver damage. The recommended screening strategy for patients over 35 years old, with hepatitis B virus (HBV) and (or) hepatitis C virus (HCV) infections, includes the determination of serum alpha-fetoprotein (AFP) levels and an abdominal ultrasound every 6 months to detect HCC at an early stage. Quantitative determination of serum AFP > 400 ng/ml lasting four weeks is valuable for the diagnosis of primary liver cancer, after excepting of active liver disease, embryonic gonad tumors and pregnancy cases [10]. However, due to low sensitivity and specificity, the clinical value of AFP is limited. In addition, AFP levels greater than 500 ng/mL are correlated with tumor size: 80% of small HCCs show no increase in AFP concentration [11]. Some patients with cirrhosis or hepatic inflammation have an elevated level of AFP without the presence of tumors [12]. Sex and features of chronic liver disease were identified as nontumor characteristics that influence serum AFP levels in patients with HCC [13]. And AFP serum levels have no prognostic meaning in well-compensated cirrhosis patients with single, small HCC treated with curative intent [14]. Therefore, it is necessary to identify new serum tumor markers to improve the early diagnosis of HCC.

Recent advances in genomics and proteomics identified a number of promising candidates which may provide superior utility over current tumor markers. Des-*γ*-carboxy prothrombin (DCP) induced by vitamin K2 absence/antagonist-II is also known as PIVKA-II (protein induced by vitamin K absence or antagonist-II). DCP is an abnormal prothrombin produced by HCC; it has completely lost the normal prothrombin function and may play an important role in the malignant proliferation of HCC. DCP is specific to HCC and less prone to elevation during chronic liver disease [15, 16]. Many studies have found that the level of serum DCP in patients with benign and malignant liver diseases is significantly different, and its diagnostic sensitivity may be higher than commonly used HCC markers such as AFP; however, this remains controversial [17, 18]. Serums DCP and AFP lack correlation and complement each other; therefore the combination of these markers may improve the diagnostic sensitivity for early HCC.

In this study, we performed a systematic review and meta-analysis to evaluate the role of DCP in the diagnosis of HCC.

## 2. Methods

### 2.1. Search Strategy

A systematic search was conducted by two investigators independently (Rong Zhu and Jing Yang). Studies were mainly searched in MEDLINE/PubMed, EMBASE, the Cochrane Central Register of Controlled Trials, CINAHL, Science Citation Index (ISI Web of Science), Chinese Biomedical Literature Database (CBM), and Chinese National Knowledge Infrastructure (CNKI) [19, 20]. In addition, the references of included articles and relevant published reports were hand searched. The search was confined to articles written in Chinese and English. No restriction was set on the year of publication. The latest search was updated in December 2012. Keywords used for the search were as follows: (1) DCP: DCP, des-*γ*-carboxy-prothrombin, des-gamma-carboxy-prothrombin, PIVKA-II, and protein induced by vitamin K absence; and (2) HCC: HCC, hepatocellular carcinoma, liver cell carcinoma, liver cancer, and hepatic cell carcinoma. Both free text and a MeSH search for keywords were employed.

### 2.2. Criteria for Selection

Articles were suitable if the following criteria were satisfied: (1) eligible studies were clinical research articles that used DCP as a serum marker for HCC; (2) the diagnosis of HCC was usually established by histopathological examination or ultrasound magnetic resonance imaging (MRI) and computer tomography (CT) when either of these techniques showed a nodule with arterial hypervascularization >2 cm [21]; (3) eligible studies should provide the sensitivity and specificity of DCP; and (4) the data were not included in a duplicate publication.

### 2.3. Criteria for Exclusion

Articles were excluded using the following criteria: (1) studies with ambiguous diagnostic criteria; (2) studies that evaluated serum DCP levels using messenger RNA, DNA, or DNA polymorphisms; (3) studies without sufficient information to make a judgment; and (4) studies that were published as reviews, letters, case reports, editorials, or comments.

### 2.4. Selection of Studies

The title and abstract of the studies based on the search results were read thoroughly to confirm eligibility and the full text of potentially eligible studies was then retrieved for further assessment. Doubts were discussed with a third investigator. The authors were contacted for further study details if necessary.

### 2.5. Data Extraction

Data were extracted from full length articles including the use of a predesigned form by two investigators (Rong Zhu and Jing Yang) independently. Disagreements were resolved by discussion. The extracted information included name of the first author, year of publication, journal, study design, diagnostic criteria, number of patients, ethnicity, type of assay used for the biomarkers, and cutoff values and raw data (the number of true positive, false positive, false negative, and true negative subjects).

### 2.6. Assessment of Methodological Quality

The quality of each study was assessed according to the QUADAS (quality assessment of studies of diagnostic accuracy included in systematic reviews) checklist recommended by the Cochrane Collaboration. Each of the 14 items in the QUADAS checklist was scored as “yes,” “no,” or “unclear” [22].

### 2.7. Indices of Diagnostic Efficacy

The indices of diagnostic efficacy included sensitivity, specificity, diagnostic odds ratio (DOR), symmetric summary receiver operating characteristic (SROC) curve, and the Q* index.

### 2.8. Data Analysis

Using the Midas model for Stata (version 11.0), funnel plots were constructed and *P* values were calculated. Publication bias existed when a *P* value < 0.05 was observed. Meta-DiSc 1.4 software was used to summarize the pooled sensitivity, specificity, PLR, NLR, and DOR and to construct a summary receiver operating characteristic (SROC) curve to calculate area under the curve. As a potential cause of heterogeneity, the threshold effect was tested using the Spearman correlation coefficient. Heterogeneity induced by other factors, such as sensitivity and specificity, was assessed using the chi-square test. PLR and NLR were assessed by Cochrane's *Q* test. Heterogeneity was investigated using the Higgins (*I* ^2^) estimate. When the *I* ^2^ value was <25%, this represented low heterogeneity; when the *I* ^2^ value was >25% and <50%, this indicated moderate heterogeneity; and when the *I* ^2^ value was >50%, this suggested high heterogeneity. The fixed effects model was used when no heterogeneity existed and the random effects model was used to collectively analyze the accuracy indicators. The results are presented with the corresponding 95% confidence intervals (CI) and the significance level *α* was 0.05. Meta-regression was also performed to explain the source of the observed heterogeneity.

## 3. Results

### 3.1. Characteristics of the Selected Studies

**Figure 1:**
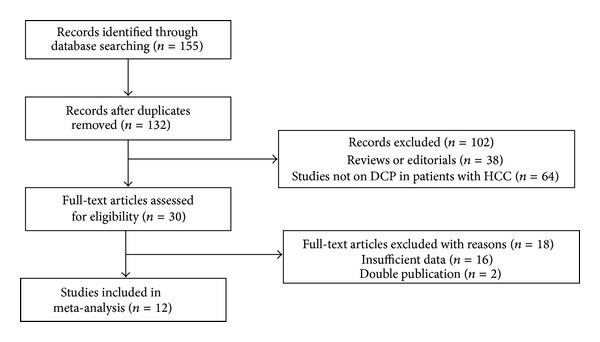
Study selection.

A total of 155 studies were identified, of which 12 [17, 23–33] were considered suitable for inclusion in the analysis after excluding summaries, case reports, duplicates, and unsuitable studies, and all were English publications. Of these 12 studies, only 2 were perspective studies [27, 33] and 10 were retrospective studies. As shown in Table 1, 12 studies involving 3,058 patients were included for meta-analysis; 1,505 of these patients had HCC and 1553 did not. A flow diagram of the study selection process is shown in Figure 1.

The characteristics of each study are shown in Table 1. The number of patients in each of the 12 studies was greater than 100, with little difference in characteristics between the studies. The DCP cutoff values in 8 studies were 40 mAU/mL [21, 23, 24, 26, 27, 30, 32, 33]. The ethnicity in 4 studies was Caucasian [27–30] and was Asian in the remaining studies.

### 3.2. Quality of the Studies

The results of the QUADAS assessment are shown in Table 2. Five studies scored A [17, 23, 30, 32, 33], 3 studies scored B [26, 27, 29], and 4 studies scored C [24, 25, 28, 31]. Various types of diseases were compared and analyzed in 8 studies [17, 23, 26, 27, 29, 30, 32, 33], while 4 other studies did not completely cover the control diseases; all studies established the gold standard (including histopathological examination and iconography evidence), which accurately distinguished between malignant and benign diseases; three studies did not supply sufficient information to determine whether blood samples were collected before the intervention [24, 26, 29]; in 7 studies the disease status was confirmed by the reference standard in all patients without the results of DCP and AFP [17, 23, 26, 29, 30, 32, 33], and another 4 studies did not provide sufficient information. Two studies did not provide an explanation as to why patients quit the trials [26, 29]. All studies provided a detailed description of the method used to determine serum DCP.

### 3.3. Results of Statistical Analysis

#### 3.3.1. Publication Bias Analysis

**Figure 2:**
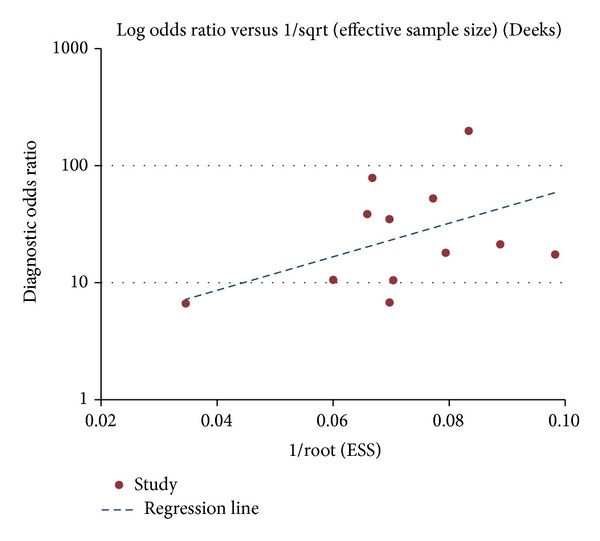
Deeks funnel plots.

Deeks funnel plots were used to examine publication bias and are shown in Figure 2. A *P* value < 0.05 showed that there was publication bias in the 12 studies.

#### 3.3.2. Heterogeneity Analysis

**Figure 3:**
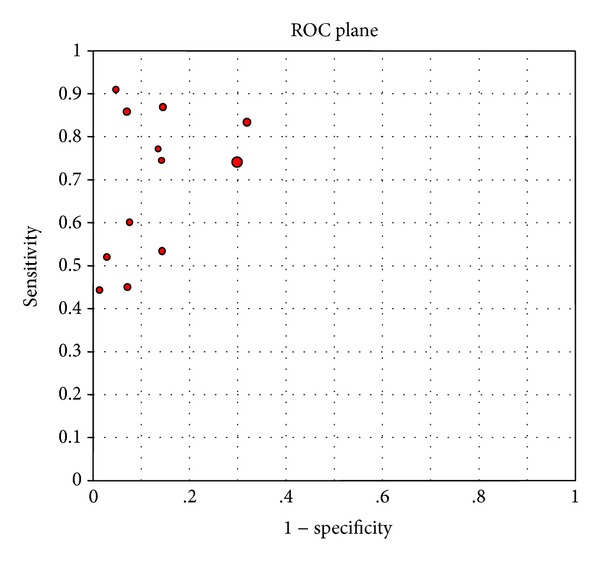
ROC scatter plot of the 12 included studies.

As differences in sensitivity, specificity, and DOR, which are caused by different cutoff values, may produce a threshold effect, it is necessary to assess the presence of a threshold effect. The ROC scatter plot would show a typical “shoulder arm” pattern and Spearman correlation analysis would show a strong positive correlation if a threshold effect existed. In this study, the ROC scatter plot obtained using Meta-DiSc 1.4 software was not the typical “shoulder arm” pattern (Figure 3). The Spearman correlation coefficient (*rs*) value was 0.336 and the *P* value was 0.286, suggesting that there was no threshold effect.

After testing for heterogeneity caused by other sources, the results showed that sensitivity (*P* = 0.000, *I* ^2^ = 93.1%), specificity (*P* = 0.000, *I* ^2^ = 92.9%), PLR (Cochrane *Q* = 98.92, *P* = 0.000, *I* ^2^ = 88.9%), NLR (Cochrane *Q* = 119.13, *P* = 0.000, *I* ^2^ = 90.8%), and DOR (Cochrane *Q* = 73.88, *P* = 0.000, *I* ^2^ = 85.1%) in the included studies showed high heterogeneity. Metaregression analysis revealed that the sources of heterogeneity were correlated with quality of the studies, type of assay used for the biomarkers, ethnicity, tumor size, and study design; however, individual factors were not associated with heterogeneity (Table 3), suggesting that the influencing factors are complex.

#### 3.3.3. Meta-Analysis

**Figure 4:**
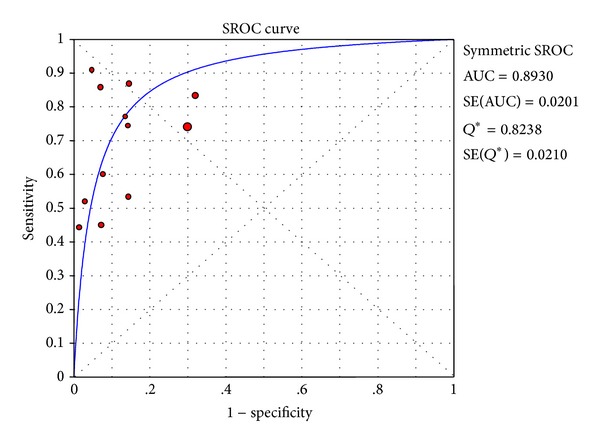
SROC of the 12 included studies.

**Figure 5:**
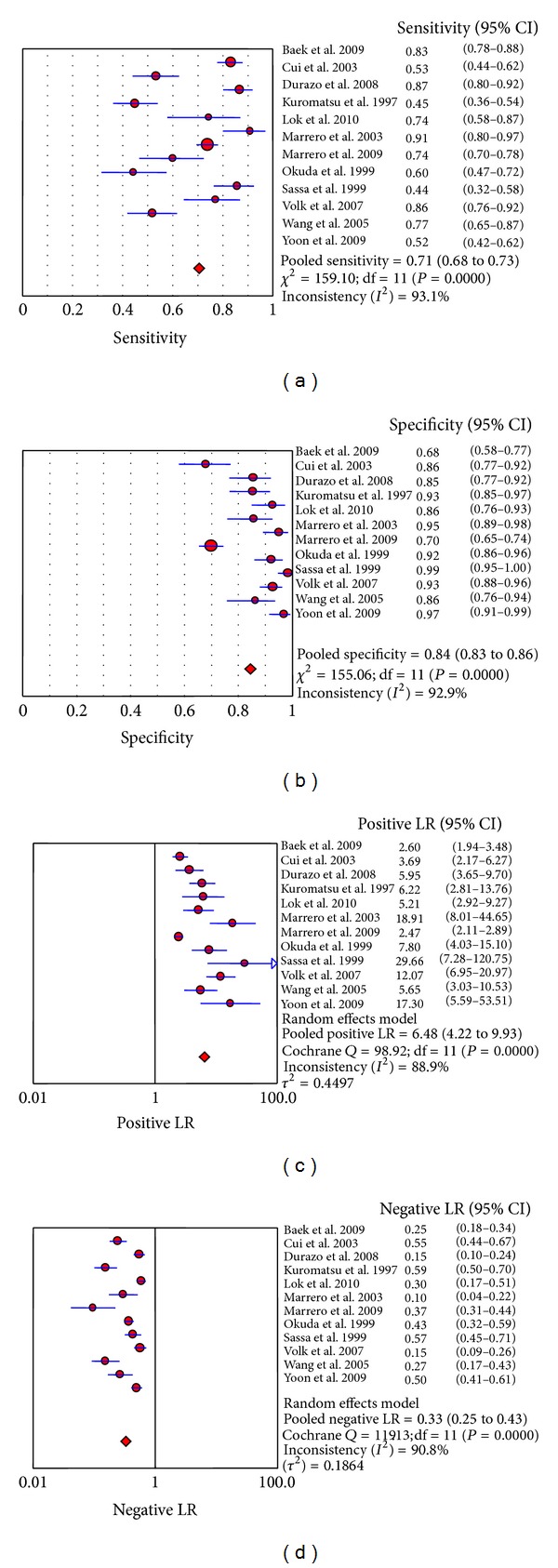
Forest map of the meta-analysis of each index: (a) sensitivity, (b) specificity, (c) PLR, and (d) NLR.

**Figure 6:**
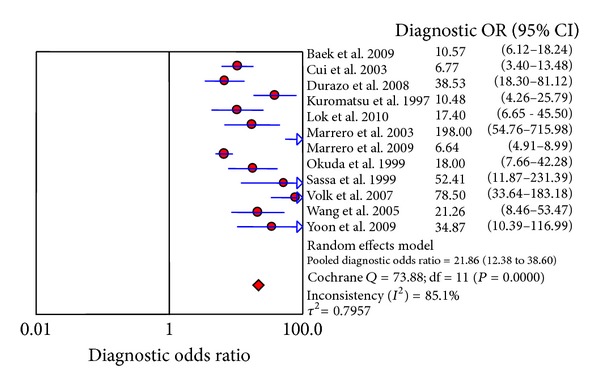
Forest map of DOR.

The DerSimonian-Laird (random effects) model was used to calculate the pooled value. The area under the curve (AUC) of the summary receiver operating characteristic curve (SROC) was 0.8930, SE = 0.0201, and Q* = 0.8238 (Figure 4). The pooled sensitivity and specificity were 71% (95%CI: 68%–73%) (Figure 5(a)) and 84% (95%CI: 83%–86%) (Figure 5(b)), respectively. The pooled PLR and NLR were 6.48 (95%CI: 4.22–9.93) (Figure 5(c)) and 0.33 (95%CI: 0.25–0.43) (Figure 5(d)) and the pooled DOR was 21.86 (95%CI: 12.38–38.60) (Figure 6), respectively.

#### 3.3.4. Sensitivity Analysis

A sensitivity analysis was carried out using the following 4 criteria to examine the stability of the meta-analysis: (1) remove 7 studies of poor quality according to the QUADAS assessment; (2) remove 3 studies which did not use ELISA detection methods; (3) patients were divided into two categories according to ethnicity: 8 studies included Asian patients and 4 studies included Caucasian patients; (4) studies included were divided into two groups: 2 perspective studies and 10 retrospective studies. The results showed that there was no significant difference in the pooled index between the 5 studies which scored A in the 9 studies which used ELISA detection methods and in the 12 studies included. In addition, these studies had overlapping confidence intervals. However, the DOR of the Caucasian studies was higher than that of the Asian studies (Asian: DOR: 17.39, AUC: 0.8761, Q*: 0.8066; Caucasian: DOR: 34.44, AUC: 0.9209, Q*: 0.8544) (Table 4).

In perspective studies and retrospective studies, there was no significant difference in DOR, but there was a difference in sensitivity and specificity.

## 4. Discussion

Early diagnosis of HCC, which is directly related to therapeutic effects and prognosis, is very important. The most commonly used screening strategy in patients with cirrhosis is the determination of serum alpha-fetoprotein (AFP) levels. However, in the majority of patients with small HCCs, the serum AFP level does not increase significantly [34, 35]. Due to the low accuracy of AFP, it is necessary to explore other serum markers with better diagnostic sensitivity and heterogeneity for HCC. Des-*γ*-carboxy prothrombin (DCP), induced by vitamin K2 absence/antagonist-II, is an abnormal prothrombin produced by HCC. DCP is specific to HCC and less prone to elevation during chronic liver disease. Therefore, DCP is a potential serum marker of HCC and may be important in the early diagnosis of HCC [36]. In this study, we attempted to review the literature and perform a meta-analysis to evaluate the role of DCP in the diagnosis of HCC.

To determine the value of using DCP as a biomarker of HCC, 12 studies fulfilling the inclusion criteria which included 3058 subjects, 1505 with HCC and 1553 without HCC, were evaluated. Heterogeneity (with the exception of the threshold effect) was found in these studies. The pooled sensitivity and specificity were 71% (95%CI: 68%–73%) and 84% (95%CI: 83%–86%), respectively. The pooled PLR and NLR were 6.48 (95%CI: 4.22–9.93) and 0.33 (95%CI: 0.25–0.43) and the pooled DOR was 21.86 (95%CI: 12.38–38.60), respectively. These results suggest that the accuracy of DCP in the diagnosis of HCC may not be as high as previously described in some studies. In the study by Marrero and colleagues [28], the sensitivity and specificity were 91% and 95%, respectively.

The likelihood ratio is a composite index of sensitivity and specificity. A LR >10 or <0.1 results in significant and conclusive shifts from pretest to posttest probability, essentially determining or excluding the diagnosis; 5 to 10 or 0.1 to 0.2 results in moderate shifts from pretest to posttest probability; 2 to 5 or 0.2 to 0.5 results in a small change in probability; 1 to 2 or 0.5 to 1 results in no change in probability [12]. The PLR in this study was 6.48, indicating that patients with HCC had more than a 6-fold higher chance of a positive DCP assay compared to patients without HCC. The PLR did not reach 10; therefore, the diagnostic accuracy of DCP for HCC was moderate. The NLR was 0.33, which indicated that if the DCP assay was negative, the probability of these patients developing HCC was approximately 33%. Thus DCP-negative results may not be used to exclude HCC.

The QUADAS tool was used to evaluate the included studies; the results showed that five studies scored A, 3 studies scored B, and 4 studies scored C, indicating that the quality of the included studies was quite different. The threshold effect according to heterogeneity tests of the included studies was not observed; sensitivity, specificity, PLR, NLR, and DOR among the included studies showed high heterogeneity, which may have been caused by the quality of the studies, type of assay used for the biomarkers, ethnicity, tumor size, study design, and other aspects. However, metaregression analysis showed that the above-mentioned factors do not significantly affect heterogeneity, suggesting that the influencing factors are complex. In the sensitivity analysis, quality of the studies and type of DCP assay had no significant effect on the results of the evaluation, and most results had overlapping confidence intervals. After dividing the 12 included studies into two categories according to ethnicity, the index in the Caucasian category was better than that in the Asian category, with the exception of specificity, indicating that the value of DCP in the diagnosis of HCC may be different between races. We guess that the differences in etiology of HCC between Asians and Caucasians may lead to this result. In Asians, chronic viral hepatitis due to hepatitis B virus (HBV) or hepatitis C virus (HCV) is the main cause of HCC; however, in Caucasians, alcoholic cirrhosis is the main cause of HCC. However, in our meta-analysis, there were only 8 studies that included Asian patients and 4 studies that included Caucasian patients; we need more clinical studies to prove this speculate.

In this study, a rigorous and rational search strategy, inclusion criteria, and statistical analyses were used to systematically and comprehensively analyze the value of serum DCP in the diagnosis of HCC. However, this study had many limitations. First, we suggest that a pathogenesis of HCC should be established and improved in the near future, to facilitate research into the molecular markers, diagnosis of HCC, and target therapies [7–9, 19, 20, 37–41]. Second, due to the poor quality of the included studies, the meta-analysis was affected by publication bias. Third, most of the studies were retrospective and the number of prospective studies was small. The number of patients with early stage HCC was not mentioned or too small to investigate the value of DCP in the diagnosis of early HCC.

## 5. Conclusions

In summary, the serum marker, DCP, showed a strong positive correlation with HCC, and the meta-analysis indicated that DCP had a moderate diagnostic accuracy for HCC. The measurement of DCP may be an optional method in the diagnosis of HCC. More studies with a rigorous design, large sample size, and multiregional cooperation are needed to obtain further evidence on the value of DCP in HCC diagnosis.

## Figures and Tables

**Table 1:** Main characteristics of the studies included in the meta-analysis.

Number	Study	TP	FP	FN	TN	*N *	Assay type	DCP cutoff value (mAU/mL)	Ethnicity	Small HCC
1	Baek et al., 2009 [23]	189	32	38	68	327	ELISA	40	Asian	No
2	Cui et al., 2003 [24]	64	13	56	77	210	EIA	40	Asian	No
3	Durazo et al., 2008 [25]	125	14	19	82	240	ELISA	84	Asian	No
4	Kuromatsu et al., 1997 [26]	58	6	71	77	212	ELISA	40	Asian	No
5	Lok et al., 2010 [27]	29	11	10	66	116	EIA	40	Caucasian	Yes
6	Marrero et al., 2003 [28]	50	5	5	99	159	ELISA	125	Caucasian	No
7	Marrero et al., 2009 [29]	310	125	109	292	836	ELISA	150	Caucasian	No
8	Okuda, 1999 [30]	36	9	24	108	177	ELISA	40	Asian	No
9	Sassa et al., 1999 [31]	27	2	34	132	195	ECL	40	Asian	Yes
10	Volk et al., 2007 [17]	72	12	12	157	253	ELISA	150	Caucasian	No
11	Wang et al., 2005 [32]	47	9	14	57	127	ELISA	40	Asian	No
12	Yoon et al., 2009 [33]	55	3	51	97	206	ELISA	40	Asian	No

TP: true positive; FP: false positive; FN: false negative; TN: true negative.

Small HCC: all tumors were ≤3 cm in diameter.

**Table 2:** Summary of methodological quality of the included studies on the basis of the review authors' judgments on the 14 items in the QUADAS checklist for each study.

QUADAS	Number											
	1	2	3	4	5	6	7	8	9	10	11	12
Representative patient spectrum?	Y	N	N	Y	Y	N	Y	Y	N	Y	Y	Y
Selection criteria	Y	Y	Y	Y	Y	Y	Y	Y	Y	Y	Y	Y
Acceptable reference standard?	Y	Y	Y	Y	Y	Y	Y	Y	Y	Y	Y	Y
Acceptable delay between tests?	Y	NR	Y	NR	Y	Y	NR	Y	Y	Y	Y	Y
Partial verification avoided?	Y	Y	Y	Y	Y	Y	Y	Y	Y	Y	Y	Y
Differential verification avoided?	Y	Y	Y	Y	Y	Y	Y	Y	Y	Y	Y	Y
Incorporation avoided?	Y	Y	Y	Y	Y	Y	Y	Y	Y	Y	Y	Y
Index test execution	Y	Y	Y	Y	Y	Y	Y	Y	Y	Y	Y	Y
Reference standard execution	Y	Y	Y	Y	Y	Y	Y	Y	Y	Y	Y	Y
Reference standard results blinded?	Y	Y	Y	Y	Y	Y	Y	Y	Y	Y	Y	Y
Index test results blinded?	Y	NR	NR	Y	NR	NR	Y	Y	N	Y	Y	Y
Relevant clinical information?	Y	Y	Y	Y	Y	Y	Y	Y	Y	Y	Y	Y
Uninterpretable results reported?	Y	Y	Y	Y	Y	Y	Y	Y	Y	Y	Y	Y
Withdrawals explained?	Y	Y	Y	NR	Y	Y	NR	Y	Y	Y	Y	Y
Quality of the studies	A	C	C	B	B	C	B	A	C	A	A	A

**Table 3:** Metaregression analysis of diagnostic accuracy.

Var.	Coeff.	Std. err.	*P* value	RDOR
Quality	−0.354	0.5196	0.5214	0.70
Assay	−1.117	1.4138	0.4596	0.33
Ethnicity	−0.625	0.8972	0.5120	0.54
Small HCC	0.994	2.0079	0.6383	2.70

**Table 4:** Results of the sensitivity analysis using 3 criteria.

Analytical perspective	Quantity	SEN (95% CI)	SPE (95% CI)	PLR (95% CI)	NLR (95% CI)	DOR (95% CI)	AUC	Q*
Included studies	12	0.71 (0.68, 0.73)	0.84 (0.83, 0.86)	6.48 (4.22, 9.93)	0.33 (0.25, 0.43)	21.86 (12.38, 38.60)	0.8930	0.8238
Studies scored A	5	0.74 (0.70, 0.78)	0.88 (0.85, 0.91)	7.06 (3.27, 15.21)	0.30 (0.20, 0.47)	24.56 (11.55, 52.23)	0.9008	0.8321
Used ELISA detection methods	9	0.73 (0.71, 0.76)	0.83 (0.81, 0.85)	6.48 (3.91, 10.73)	0.29 (0.20, 0.41)	24.29 (12.11, 48.70)	0.9001	0.8313
Ethnicity								
Asian	8	0.66 (0.63, 0.69)	0.89 (0.86, 0.91)	6.16 (3.83, 9.91)	0.39 (0.29, 0.52)	17.39 (10.61, 28.51)	0.8761	0.8066
Caucasian	4	0.77 (0.74, 0.81)	0.80 (0.77, 0.83)	7.06 (2.54, 19.63)	0.22 (0.12, 0.40)	34.44 (7.02, 168.96)	0.9209	0.8544
Type								
Perspective	2	0.58 (0.49, 0.66)	0.92 (0.87, 0.96)	8.78 (2.30, 33.43)	0.41 (0.24, 0.69)	22.77 (10.72, 48.33)	0.5000	0.5000
Retrospective	10	0.72 (0.69, 0.74)	0.84 (0.81, 0.85)	6.17 (3.90, 9.76)	0.31 (0.23, 0.43)	21.62 (11.41, 40.99)	0.8875	0.8181

SEN: sensitivity; SPE: specificity; PLR: positive likelihood ratio; NLR: negative likelihood ratio; DOR: diagnostic odds ratio; AUC: area under curve.
